# Identifying differential correlation in gene/pathway combinations

**DOI:** 10.1186/1471-2105-9-488

**Published:** 2008-11-18

**Authors:** Rosemary Braun, Leslie Cope, Giovanni Parmigiani

**Affiliations:** 1 National Cancer Institute, National Institutes of Health, Bethesda, MD 20892, USA; 2 The Sydney Kimmel Comprehensive Cancer Center, Johns Hopkins University, Baltimore, MD 21205, USA

## Abstract

**Background:**

An important emerging trend in the analysis of microarray data is to incorporate known pathway information a priori. Expression level "summaries" for pathways, obtained from the expression data for the genes constituting the pathway, permit the inclusion of pathway information, reduce the high dimensionality of microarray data, and have the power to elucidate gene-interaction dependencies which are not already accounted for through known pathway identification.

**Results:**

We present a novel method for the analysis of microarray data that identifies joint differential expression in gene-pathway pairs. This method takes advantage of known gene pathway memberships to compute a summary expression level for each pathway as a whole. Correlations between the pathway expression summary and the expression levels of genes not already known to be associated with the pathway provide clues to gene interaction dependencies that are not already accounted for through known pathway identification, and statistically significant differences between gene-pathway correlations in phenotypically different cells (e.g., where the expression level of a single gene and a given pathway summary correlate strongly in normal cells but weakly in tumor cells) may indicate biologically relevant gene-pathway interactions. Here, we detail the methodology and present the results of this method applied to two gene-expression datasets, identifying gene-pathway pairs which exhibit differential joint expression by phenotype.

**Conclusion:**

The method described herein provides a means by which interactions between large numbers of genes may be identified by incorporating known pathway information to reduce the dimensionality of gene interactions. The method is efficient and easily applied to data sets of ~10^2 ^arrays. Application of this method to two publicly-available cancer data sets yields suggestive and promising results. This method has the potential to complement gene-at-a-time analysis techniques for microarray analysis by indicating relationships between pathways and genes that have not previously been identified and which may play a role in disease.

## Background

Advances in microarray technology have permitted the monitoring of gene expression in cells with known phenotypic differences. These experiments commonly produce data sets containing expression levels of tens of thousands of genes for tens or hundreds of samples, and thus the analysis of such high-dimensional data is of considerable interest.

In the most basic analyses, two sets of data (e.g., from disease and normal tissue) are examined for differential gene expression though statistical testing (including *t*-tests and empirical Bayes approaches) followed by multiple-comparison corrections. Common testing techniques have been reviewed [1,2]. A drawback to these methods results from the fact that each gene is examined individually, despite the fact that there exist well-established biological relationships between genes; typically, pathway information is incorporated after differentially expressed genes have been identified (as reviewed [3]). Multi-gene effects are often contemplated through the use of cluster analysis [4-6], which attempts to identify associated groups of genes, or gene-set enrichment analyses [7,8], which identifies sets in which differentially expressed genes are over represented. As with single-gene approaches, gene interactions for which the marginal distributions of the individual genes are similar may be missed by these analyses.

Recent work [9,10] addresses this common drawback by examining the expression of gene pair combinations and identifying gene pairs for which the joint association differs in two phenotypes. Dettling and coworkers [10] propose a scoring function to flag differential correlation between genes; for instance, situations in which the two genes show correlated expression in normal cells but show anti-correlated expression in tumor cells would be noted, despite the fact that the marginal distributions of the individual gene expression levels may be indistinguishable. In contrast to the cluster and enrichment analysis techniques mentioned above, the analysis is not restricted to single differentially expressed genes; rather, all possible gene pairs are explored for phenotype-related dependencies and interactions. This method, which showed promising results on several datasets [10], has the power to suggest heretofore unknown interactions between gene pairs which may have biological relevance in the phenotypes of interest.

In this work, we expand the aforementioned techniques [9,10] to incorporate existing biological knowledge by considering known pathways rather than individual genes. In order to reduce the dimensionality of the problem, we employ principal component analysis to define a summary expression level for the genes known to be involved in a given pathway. The method presented here searches for gene-pathway pairs for which a phenotype-conditional correlation exists between the gene expression level and the pathway summary expression level. Measures of the reliability of the pathway summary expression level are obtained, and significance of the phenotype-conditional correlation differences is assessed through permutation testing.

A related analysis has been proposed by Li [11-13], which searches for pairs of genes (or two orthogonal projections of a gene set) that are mediated by a third gene. Ho and collaborators showed [11] that if the mediating variable is binary (e.g., representing phenotype rather than disease expression), the Liquid Association score proposed by Li is formally equivalent to the correlation score propsed in [10]. The method we propose applies similar mathematical principles to an independent problem, namely, finding gene-pathway pairs which are driven by phenotype.

In this paper, we detail the methodology illustrated above and apply it to a public-domain gene expression data set consisting of normal and tumor prostate cell samples [14] as well as to gene expression data from lung adenocarcinoma and squamous cell carcinoma [15]. Several promising results are obtained for genes that were not previously identified as having differential expression in the normal and tumor samples, suggesting that this novel analysis technique has the potential to reveal new interactions. Because of the efficiency and scalability of this technique, it is well suited to the large data sets produced in modern microarray experiments.

## Results and discussion

### Algorithm

We wish to identify gene-pathway pairs (*G*, *P*) for which there exists a pronounced difference in association between phenotypes. In order to reduce the dimensionality of the pathway data, we employ principal component analysis to define a one-dimensional summary *p*_*k *_of the expression values of the genes in the pathway *P *for sample *k*. Relationships between pathway summary expressions *p *and individual gene expressions *g *(for which the gene is not already a known member of the pathway) may then be compared between two phenotypes. This method has the advantage of succinctly accounting for expression levels across whole pathways, and has the potential to indicate interactions between genes and pathways that have not yet been identified.

**Figure 1:**
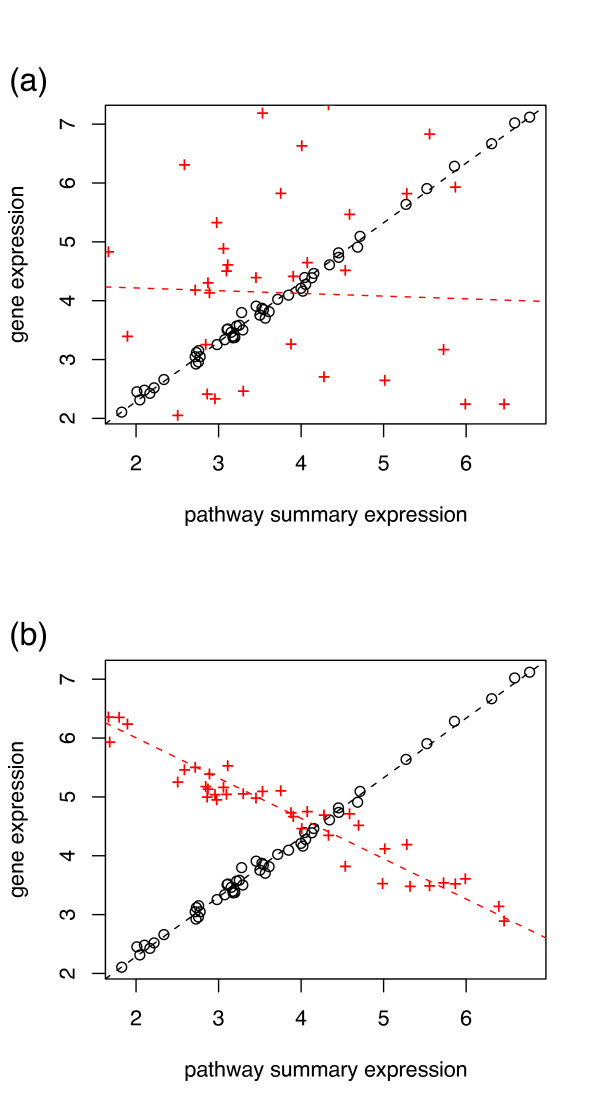
**Joint differential expression examples**. Example loss-of-correlation plots. Red crosses indicate one hypothetical phenotype; black circles, another hypothetical phenotype. (a) loss of correlation; (b) a reversal of correlation.

Simulated cases of interest are illustrated in Fig. 1. Here, the *x*-axis is the summary expression level for the pathway as a whole (*p*), and the *y*-axis is the expression level for the gene (*g*). Two different situations are depicted: in the top figure, a strong correlation in gene and pathway expression in the first phenotype is lost in the second phenotype; and in the bottom figure, a strong positive correlation in gene and pathway expression in the first phenotype is replaced with an anticorrelation in the second phenotype. Biologically, such cases could arise in situations where the gene plays a role related to a pathway, and for which the alteration of this interaction affects the phenotype.

We identify cases such as those illustrated in Fig. 1 by computing a simple score for each gene-pathway pair, using a measure of pairwise dependence *ρ*(*g*, *p*) among the gene and pathway-summary expressions. By restricting this measure to just the samples from each phenotype and obtaining class-conditional correlations *ρ*_0_(*g*, *p*), *ρ*_1_(*g*, *p*) for phenotypes 0 and 1 respectively, we can define the Gene-Pathway Correlation Score (GPC-score, *S*_GPC_) as the absolute value of difference between these:

(1) *S*_GPC_(*g*, *p*) = |*ρ*_1_(*g*, *p*) - *ρ*_0_(*g*, *p*)|, *g *∉ *G*_*P*_,

where *G*_*P *_is the set of all genes in the pathway of interest. The gene-pathway pairs (*g*, *p*) which lie at the high end of the distribution are flagged as potentially similar to the examples in Fig. 1. For a given pair, it is possible to perform a permutation test to assess the probability that the observed score *S*_GPC_(*g*, *p*) would have appeared had there been no association between the correlations *ρ *and the phenotypes.

### Implementation

Here we detail the critical steps of the algorithm: selection and summarization of pathways, computation of *S*_GPC_(*g*, *p*), and identification and significance testing of high-scoring pairs. The steps were implemented using the R language for statistical computing [16,17], and an R package called GPCscore containing the necessary functions is available [see Additional file 1].

#### Pathway summarization

Annotations from the Kyoto Encyclopedia of Genes and Genomes (KEGG [18]) were used to associate the genes with known pathways. For each pathway, we define the pathway expression summary as the first principal component of the pathway genes' expressions, computed from the matrix of expression values of the genes included in that pathway. For sample *k*, the projection of the gene expression data along the first principal component provides a single value *p*_*k *_which we use as the summary expression level for the pathway in sample *k*.

Principal component analysis (PCA) is a dimension reduction technique that produces a set of independent axes (principal components) as linear combinations of the original variables such that the greatest variance in the data comes to lie on the first axis (the first principal component), the second greatest variance along the second principal component, and so forth [19]. In practice, the principal component basis set is computed by singular value decomposition of the data. This permits the majority of the variation of a set of coordinates (here, expression levels of the genes in the pathway) to be summarized by the lowest order principal components, thus reducing dimensionality in a dataset while retaining those characteristics that most contribute to the variance. An alternative, but less numerically stable approach is to perform eigen decomposition of the covariance matrix; the variance projected along each component is given by the square of the eigenvalue. A complete discussion of PCA may be found in [19,20].

PCA has recently been proposed as a means by which to assess collinearity in pre-defined gene sets [21] by computing the number of principal components required to capture a given threshold of variance. Here, we exploit the putative collinearity in gene pathways: for each pathway defined by KEGG, the first principal component of the expression of the participating genes (PC1) was computed and kept as a summary of the expression of the genes comprising that pathway. Pathways for which the PC1 was not a meaningful descriptor of the overall activity were excluded at this point, as described below.

#### Pathway inclusion criteria

Because of the information loss inherent in reducing the expression levels of a collection of genes to a single figure, pathways for which the proportion of variance carried by the PC1 was less than an arbitrarily set threshold were considered to be inadequately described by the PC1 alone and excluded. In practice we required the proportion of variance carried by the PC1 to exceed 0.60.

Additionally, the (normalized) phenotype-conditional principal component basis vectors were checked for comparability by using a dot-product. Those with non-parallel class-conditional PC1s were flagged as having within-pathway differences resulting from differential expression of some subset of pathway components. While such cases may be of biological interest, these pathways could not be meaningfully compared on a common basis, and so were excluded from further analysis by this technique.

The minimum dot-product threshold was chosen by simulating the distribution of pathway PC1 dot-products in phenotypically similar samples. Specifically, the data from 50 normal prostate tissue samples was split at random into two groups, and the PC1 dot-products for each pathway were computed between the two groups; this resampling was carried out 10^3 ^times. It was found that fewer of than 5% (0.042) of resampled dot-products fell below 0.9, suggesting that 0.9 is a reasonable expectation of parallelism.

Pathways with compatible basis vectors (*i.e*., those for which the class-conditional PC1 basis vectors had dot-products exceeding 0.9) were retained for further analysis, and the projection of each sample's expression data onto the first principal component of the pathway was computed, thus providing a summary expression level *p*_*k *_in each sample *k *for each pathway *P *.

#### Gene-Pathway correlations

To address phenotype-related differences in gene-pathway interactions, correlations between gene expression levels and pathway summary expression levels were examined.

For each gene-pathway pair, Spearman's rank correlation was computed separately within each phenotype, and the absolute value of the difference between phenotypes *S*_GPC_(*g*, *p*) (Eq. 1) was considered. Spearman correlation is not strongly influenced by outlying samples, and has the benefit of being invariant to monotonic transformations of the data. Pairs for which the gene under consideration was also an element of the pathway were excluded, as our interest lies in the interplay between pathways and genes not already known to be associated with the pathway. Additionally, to limit the number of pairs for which high *S*_GPC _is attributable to strong diffrential expression in gene *g*, pairs in which the gene of interest has a univariate *t*-test FDR exceeding a user-set threshold may be excluded.

**Figure 2:**
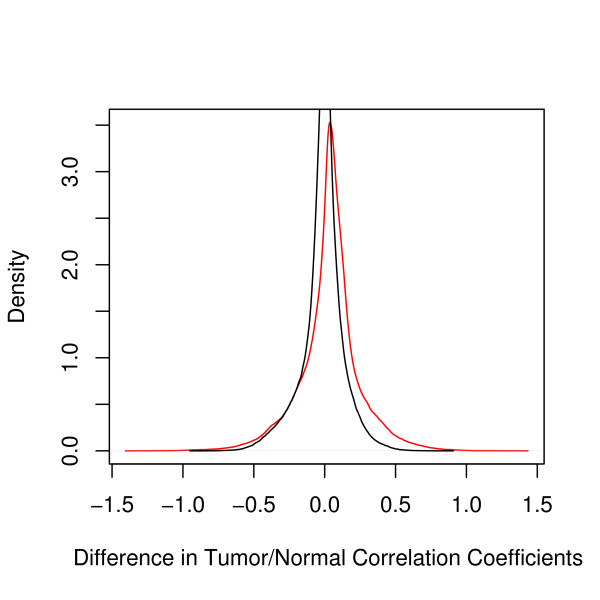
**Distribution of gene-pathway correlation loss, prostate data**. Distribution of loss of correlation between normal and tumor samples (ie, signed *S*_GPC_) in the prostate data set (red) and 6 permutation resamplings (black) of all pairs. While not exhaustive, the few resamplings show a much narrower distribution centered around zero; none of the resampled pairs exhibited values exceding *S *= 0.9. By contrast, the observed distribution is wider, and biased towards loss of correlation in tumor samples with respect to normal samples.

Gene-pathway pairs meeting the above criteria which had large *S*_GPC_(*g*, *p*) values were chosen as relevant. The distribution of *S*_GPC_(*g*, *p*) in the prostate data may be seen in Fig. 2. In addition, we required flagged pairs to have a gene-pathway correlation approaching what would be expected of an interacting pair in at least one of phenotypes, as described below.

#### Pathway Coherence

The GPC-score is motivated by the reasoning that if a gene *g *interacts with a pathway *P*, it will exhibit a high correlation with the summary expression level for that pathway in biologically normal cells. We examined the strength of this assumption by taking known pathways, treating a gene *g*_*P *_of that pathway as "unknown," calculating the pathway summary expression level $p_{\neg g_{P}}$ for the pathway *without g*_*P*_, and then computing a "within-pathway correlation"

(2) $$\begin{array}{cc} \rho (g_{P},p_{\neg g_{P}}), & g_{P}\in G_{P} \end{array}$$

where *G*_*P *_is the set of all genes comprising the pathway. We expect that the distribution of |*ρ *(*g*_*P*_, $p_{\neg g_{P}}$)| is high relative to that of |*ρ *(*g*, *P*)|, *g *∉ *G*_*P*_; indeed, a nonparametric (Wilcoxon rank-sum) test using the normal prostate data revealed a significantly higher (*p *< 2.2·10^-16^) location of the in-path correlations versus the out-of-path correlations.

**Figure 3:**
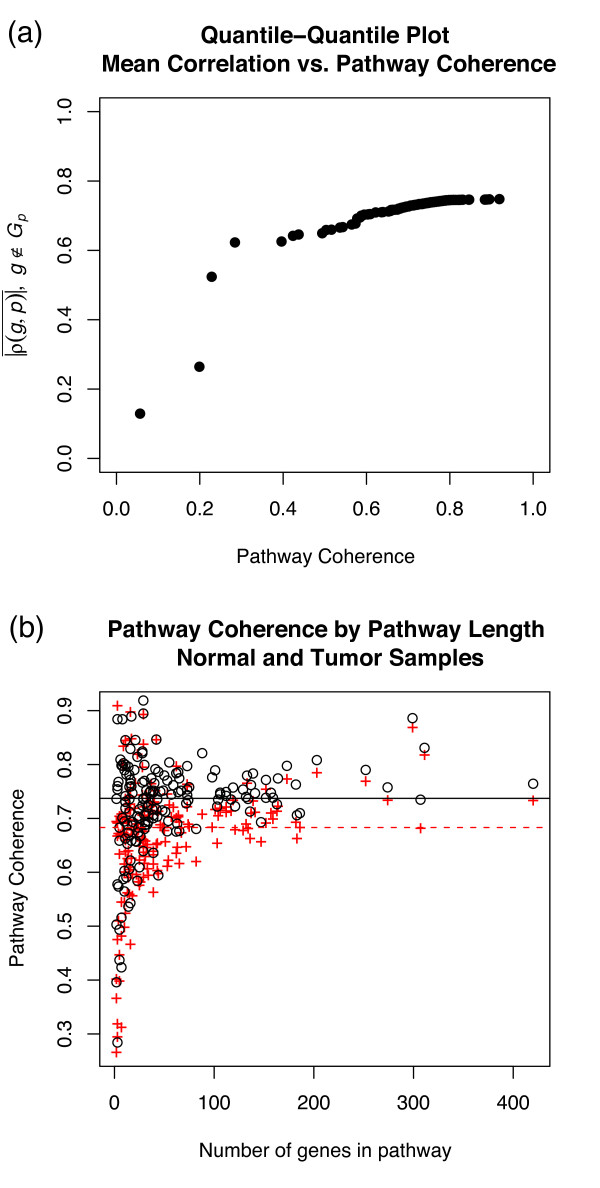
**Pathway coherence in normal prostate samples**. (a) Q-Q plot of mean correlation across all genes for each pathway vs. coherenece for each pathway. Pathway coherence has a much broader distribution; in particular, pathway coherence exceeding 0.7 is much more common than the average gene-pathway correlation (across all genes not on the pathway). (b) Pathway coherence vs. pathway length for normal prostate (black circles) and prostate tumor (red crosses) samples; pathway coherence is systematically lower in tumor samples.

We can define the "pathway coherence" *C*_*P *_as the average absolute value of the within-path correlations

(3) $$\begin{array}{cc} C_{P}=\bar{\left|\rho (g_{P},p_{\neg g_{P}}\right|}, & g_{P}\in G_{P}, \end{array}$$

where the bar denotes an arithmetic mean across all genes in the pathway. We expect that, for most pathways, the coherence is high relative to a similar average of *ρ*(*g*, *p*) across genes unrelated to the pathway, as shown in Fig. 3a. To ensure biologicaly representivity, it is best to measure pathway coherence in data from normal or wildtype tissue; indeed, the pathway coherence is systematically lower in tumor tissue in both data sets tested, as illustrated in Fig. 3b.

The distribution of *ρ*(*g*_*P*_, $p_{\neg g_{P}}$) within a given pathway is used to select high *S*_GPC _pairs such that the correlation *ρ*(*g*, *p*) in one of the phenotypes is similar to or stronger than the correlations exhibited by genes already known to play a role in that pathway. In practice, this is achieved by computing the quantile of |*ρ *(*g*_*P*_, $p_{\neg g_{P}}$)| in which |*ρ *(*g*, *p*)| would fall and setting a threshold quantile above which |*ρ *(*g*, *p*)| exhibits sufficiently strong correlation to be considered a likely pathway candidate.

#### Significance testing

Once pairs of interest are selected, the significance of the phenotype-conditional correlation difference *S*_GPC_(*g*, *p*) for a given gene-pathway pair may be assessed via permutation. By constructing data subsets that include only the genes of interest (the chosen gene and those on the pathway), resampled computations of *S'*_GPC _(*g*, *p*) under random permutations of the phenotype labels can be performed in a targeted way with relatively small memory requirements and computational overhead. The permutation replicates are likewise subject to the constraint that the class-conditional PC1 vectors be parallel, and replicates that fail this are not counted; i.e., the permutation provides the null distribution of *S*_GPC _given that the class-conditional pathway projections are comparable.

Because of the large number of pairs (~10^6^), a resampling of all gene-pathway pairs is possible only for a relatively small number of complete sets; however, this is sufficient to estimate a null distribution of *S*_GPC_(*g*, *p*). In our applications, this is much narrower than the observed distribution (cf Fig. 2). We estimate that the probability of observing a value ${S^{\prime }}_{\text{GPC}}$(*g*, *p*) > 0.86 to be less than 10^-6^. As expected, the resampled distribution peaks at ${S^{\prime }}_{\text{GPC}}$(*g*, *p*) = 0.000, while the peak is at *S*_GPC_(*g*, *p*) = 0.040 for the observed distribution.

### Application and Testing

The utility of the described method is illustrated by application to two publicly-available data sets. The first data set comprises 52 tumor and 50 normal prostate tissue samples hybridized to Affymetrix HG-U95A chips [14], providing expression levels for 12625 genes. Data were normalized using gc-RMA [17,22] and expressed on a log_2 _scale. The second is a lung cancer data set of 160 samples, of which 139 samples were adenocarcinoma and 21 samples were squamous cell carcinoma [15]. As with the prostate data, the lung data were also derived from Affymetrix HG-U95A chips monitoring 12625 genes; in this case, data were normalized using RMA [17] and expressed on a log_2 _scale.

The KEGG pathway database [18] was used to establish biologically related gene subsets as described above. Of the 177 pathways identified from the genes represented on these arrays, 4 were trivial (i.e., had only a single probe represented) and were eliminated from further analysis; of the 173 remaining, the median number of genes per pathway was 27, and the maximum was 386.

For each pathway, the first principal component basis vector (PC1) was computed conditioned on phenotype. Because we are primarily interested in gene-pathway pairs which exhibit joint differential expression between the two phenotypes, it is necessary to ensure that the pathway PC1s are comparable between the two phenotypes; the two class-conditional PC1s were considered sufficiently parallel for dot products with an absolute value ≥ 0.9, as described above. In the prostate cancer data set, 11 pathways exhibited nonparallel PC1 vectors; in the lung cancer data set, 50 did. As a further criterion, only those pathways for which the first principal component represented 60% or more of the total variance were considered in further analysis (pathways with less were considered poor candidates for summarization by a single value).

In the prostate cancer data set, 81 passed this criterion; in the lung cancer data set, only 38 did.

Projections of each sample's gene expression profile onto the PC1 for the remaining pathways were computed, resulting in pathway expression summary values. Correlation coefficients for gene-pathway pairs were computed as column-wise operations on two matrices (genes vs. samples and paths vs. samples) for each phenotype, and the GPC-Score *S*_GPC_(*g*, *p*) (Eq. 1) was calculated. The computation of all *S*_GPC _values (after normalization) requires under 300 CPU-seconds, of which 10% was system time, in an interactive R [16,17] session on a 1.5 GHz PowerPC G4 with 768 MB memory.

The overall reduction in gene-pathway correlation amongst the prostate tumor samples with respect to the normal prostate samples can be seen in the density plots given in Fig. 2. Gene-pathway pairs were selected on the basis of high *S*_GPC_(*g*, *p*), excluding pairs for which gene *g *is known to be a member of pathway *P*. (Differential pathway-summary expression levels between phenotypes may be assessed using a *t*-test in a manner analagous to examining differential gene expression. Bonferroni-adjusted *p *values for six nontrivial paths with significant differential summary expression in the prostate data set are given in Table 1; however, we focus in this paper on gene-pathway pairs rather than differentially expressed pathways.) Gene-pathway pairs for which the expression correlation in one of the phenotypes was above the median of the within-pathway correlation distribution were considered likely candidates for a gene-pathway interaction (as described above). Of those, the gene-pathway pairs with the highest *S*_GPC _values from each data set are summarized in Tables 2 and 3 for the prostate and lung data, respectively.

**Table 1:** Differential pathway expression

KEGG ID	Pathway description	*N *(genes)	*p*
00061	Fatty acid biosynthesis	6	10^-5^
00510	N-Glycan biosynthesis	33	0.019
00460	Cyanoamino acid metabolism	10	0.021
00430	Taurine and hypotaurine metabolism	10	0.023
00642	Ethylbenzene degradation	14	0.038
00930	Caprolactam degradation	11	0.045

Pathways with differential PC1 expression levels between prostate normal and prostate tumor tissue at *p *< 0.05 (unpaired *t*-test).

**Table 2:** High *S*_GPC _pairs, prostate

Affy HGU95 ID	Gene symbol	KEGG ID	Pathway description	*S*_GPC_	*p*
39637_at	SLC26A2	00300	Lysine biosynthesis	0.928	<1e-04
39799_at	FABP5	00300	Lysine biosynthesis	0.829	<1e-04
39794_at	USP8	00300	Lysine biosynthesis	0.824	<1e-04
40773_at	MYL5	00300	Lysine biosynthesis	0.82	<1e-04
40027_at	ATP5S	00300	Lysine biosynthesis	0.814	1e-04
32001_s_at	PCSK6	04742	Taste transduction	0.807	<1e-04
32001_s_at	PCSK6	00600	Sphingolipid metabolism	0.799	<1e-04
32001_s_at	PCSK6	05110	Cholera – Infection	0.798	1e-04
41275_at	E2F5	00300	Lysine biosynthesis	0.798	3e-04
32001_s_at	PCSK6	00604	Glycosphingolipid biosynthesis – ganglioseries	0.786	<1e-04
32001_s_at	PCSK6	00061	Fatty acid biosynthesis	0.771	<1e-04
32001_s_at	PCSK6	00290	Valine, leucine and isoleucine biosynthesis	0.771	<1e-04
39945_at	FAP	00300	Lysine biosynthesis	0.768	6e-04
32001_s_at	PCSK6	03060	Protein export	0.765	<1e-04
38571_at	FGFR1OP	04740	Olfactory transduction	0.76	<1e-04
32001_s_at	PCSK6	00190	Oxidative phosphorylation	0.751	<1e-04
38571_at	FGFR1OP	00440	Aminophosphonate metabolism	0.749	<1e-04
40176_at	TRIM27	00625	Tetrachloroethene degradation	0.742	<1e-04
38571_at	FGFR1OP	04630	Jak-STAT signaling pathway	0.739	<1e-04
38571_at	FGFR1OP	04020	Calcium signaling pathway	0.737	<1e-04
38571_at	FGFR1OP	00150	Androgen and estrogen metabolism	0.734	<1e-04
38571_at	FGFR1OP	04930	Type II diabetes mellitus	0.733	<1e-04
32001_s_at	PCSK6	00930	Caprolactam degradation	0.732	1e-04
38571_at	FGFR1OP	04340	Hedgehog signaling pathway	0.73	3e-04
38571_at	FGFR1OP	00860	Porphyrin and chlorophyll metabolism	0.728	2e-04
32001_s_at	PCSK6	00903	Limonene and pinene degradation	0.728	<1e-04
38571_at	FGFR1OP	04650	Natural killer cell mediated cytotoxicity	0.724	2e-04
38571_at	FGFR1OP	04810	Regulation of actin cytoskeleton	0.721	1e-04
38571_at	FGFR1OP	01510	Neurodegenerative Disorders	0.721	<1e-04
40925_at	C7orf44	00300	Lysine biosynthesis	0.72	1e-04
38571_at	FGFR1OP	04660	T cell receptor signaling pathway	0.719	<1e-04
861_g_at	MSH2	03020	RNA polymerase	0.713	<1e-04
38571_at	FGFR1OP	00903	Limonene and pinene degradation	0.712	<1e-04
38571_at	FGFR1OP	00290	Valine, leucine and isoleucine biosynthesis	0.711	2e-04
38571_at	FGFR1OP	04920	Adipocytokine signaling pathway	0.71	<1e-04
38571_at	FGFR1OP	00930	Caprolactam degradation	0.708	0.0013
32001_s_at	PCSK6	00471	D-Glutamine and D-glutamate metabolism	0.707	<1e-04
38571_at	FGFR1OP	00140	C21-Steroid hormone metabolism	0.706	1e-04
38571_at	FGFR1OP	00562	Inositol phosphate metabolism	0.704	<1e-04
38571_at	FGFR1OP	00604	Glycosphingolipid biosynthesis – ganglioseries	0.702	7e-04

High-ranking gene-pathway pairs in prostate data, sorted by descending *S*_GPC_. Affymetrix ID, gene symbol, KEGG pathway ID, and pathway description are given, along with the GPC scores; *p *values are based on a permutation test with 10^4 ^resamplings.

**Table 3:** High *S*_GPC _pairs, lung

Affy HGU95 ID	Gene symbol	KEGG ID	Pathway description	*S*_GPC_	*p*
38835_at	TM9SF1	00900	Terpenoid biosynthesis	0.621	5e-04
41129_at	TMEM41B	00900	Terpenoid biosynthesis	0.603	0.0017
39405_at	UTP14C	00900	Terpenoid biosynthesis	0.58	0.0015
32150_at	GOLGA4	00900	Terpenoid biosynthesis	0.558	0.0049
1394_at	RHOA	00900	Terpenoid biosynthesis	0.534	0.0039
326_i_at	RPS20*	03010	Ribosome	0.507	0.0015
39009_at	LSM3	03050	Proteasome	0.484	0.0053
37748_at	KIAA0232	00900	Terpenoid biosynthesis	0.477	0.0022
33859_at	SAP18	00900	Terpenoid biosynthesis	0.463	0.0072
31853_at	EED	03050	Proteasome	0.462	0.0105
38668_at	GPATCH8	00900	Terpenoid biosynthesis	0.461	0.0064
40140_at	RNF103	00900	Terpenoid biosynthesis	0.456	0.0077
34326_at	COPB1	00900	Terpenoid biosynthesis	0.45	0.0083
33880_at	ACSL3	00900	Terpenoid biosynthesis	0.441	0.0119
40623_at	UBE3B	00900	Terpenoid biosynthesis	0.422	0.0093
33423_g_at	SEC13	03050	Proteasome	0.411	0.0089
37891_at	YIPF6	00900	Terpenoid biosynthesis	0.408	0.0105
41159_at	CLTC	00900	Terpenoid biosynthesis	0.408	0.0193
35845_at	SEC24B	00900	Terpenoid biosynthesis	0.405	0.0179
32051_at	ALG8	00900	Terpenoid biosynthesis	0.397	0.0139
39336_at	ARF3	00900	Terpenoid biosynthesis	0.385	0.0131
40115_at	ATP5C1	03050	Proteasome	0.384	0.0138
38811_at	ATIC	03050	Proteasome	0.378	0.0188
40411_at	NCOA6	00900	Terpenoid biosynthesis	0.377	0.0117
1985_s_at	NME1	03050	Proteasome	0.369	0.0073
35760_at	ATP5H	03050	Proteasome	0.356	0.0178
35290_at	YTHDF3	00900	Terpenoid biosynthesis	0.348	0.0033
40605_at	SNX4	00900	Terpenoid biosynthesis	0.348	0.0163

High-ranking gene-pathway pairs in lung data, sorted by descending *S*_GPC_. Affymetrix ID, gene symbol, KEGG pathway ID, and pathway description are given, along with the GPC-scores; *p *values are based on a permutation test with 10^4 ^resamplings. *The 326_i_at-Ribosome pair was included in the analysis due to the absence of annotation data for 326_i_at, but is correcly identified by the analysis as related; see the Results section.

#### High SGPC pairs in sample data sets

##### Prostate data

In the prostate cancer data set, 867492 gene-pathway pairs were eligible for inclusion using the criteria laid out above, with median *S*_GPC _of 0.09 and *S*_GPC _values over 1 falling in the 0.999-th quantile of the distribution; the median loss of correlation between the normal and tumor sets (i.e., without taking the absolute value in Eq. 1) was 0.03, indicating that the correlations tend to be higher in the normal samples than in the tumor samples.

**Figure 4:**
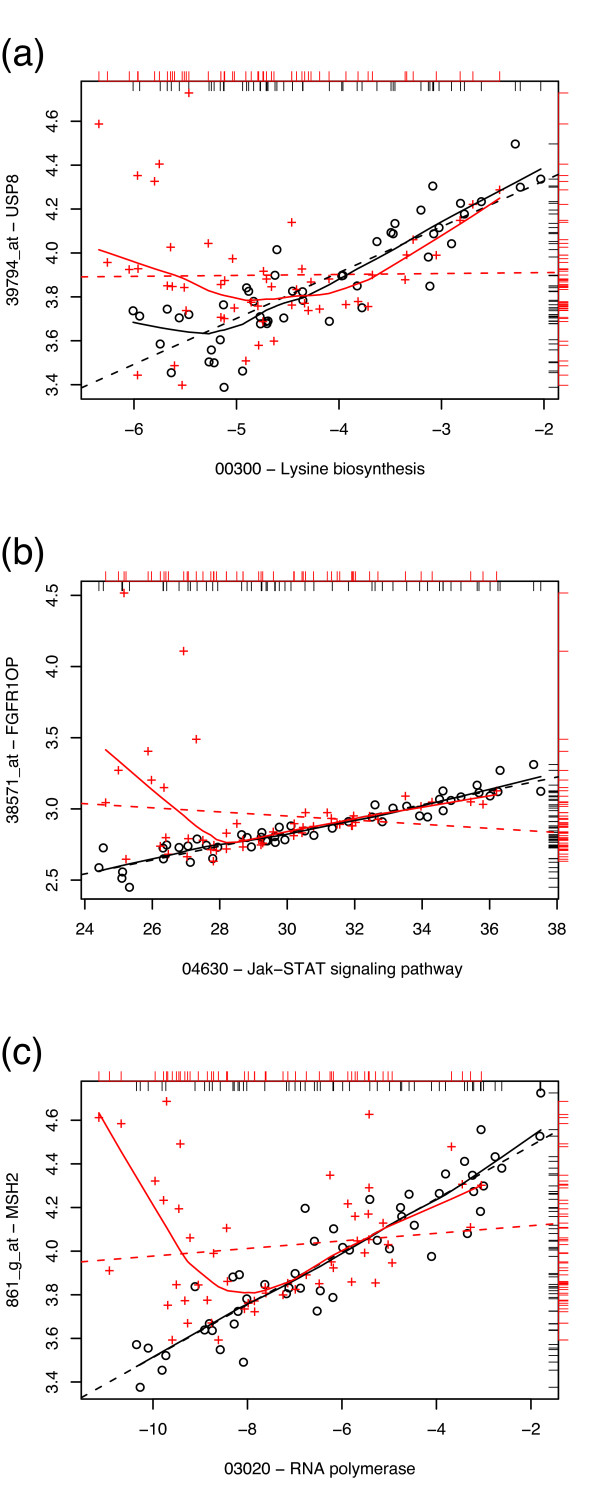
**High *S *pairs, prostate data**. Sample plots of gene expression vs. pathway expression in normal (black circles) and tumor (red crosses) prostate samples. Loess curves (solid line) and least squares linear fits (dashed line) for the two classes are given as a visual guide. Marginal distributions of the data are given as rug plots (black, inward for normal samples; red, outward for tumor samples). From top to bottom: (a) ubiquitin specific peptidase 8 (USP8) vs. lysine biosynthesis pathway (*S*_GPC _= 0.824); (b) FGFR1 oncogene partner (FGFR1OP) vs. Jak-STAT signaling pathway (*S*_GPC _= 0.739); (c) mutS homolog 2, colon cancer (MSH2) vs. RNA polymerase pathway (*S*_GPC _= 0.713).

At the high-*S*_GPC _end of the distribution in the prostate data, the gene-pathway pairs tend to exhibit similar loss-of-correlation patterns between the tumor and normal phenotype; in most cases, a strong correlation between the gene and the pathway is lost in the tumor samples, particularly at low values of gene expression. In all flagged cases, a subset of the tumor samples behave as normal. The decorrelated tumor samples almost always result from tumor gene expressions above (rarely below) the normal gene expressions. Sample gene vs. pathway expression plots are given in Fig. 4 to illustrate this scenario. For the flagged pairs listed in Table 2, *p*-values were obtained through 10^4 ^random permutations of the tumor/normal labels; for the majority of flagged pairs, none of the 10^4^permutations produced a difference in correlation values greater than or equal to the observed *S*_GPC_, suggesting *p *< 10^-4 ^with an accuracy (based on the binomial 99% confidence interval) of 5.3 · 10^-4^; the highest *p*-value obtained was *p *= 0.0013.

**Figure 5:**
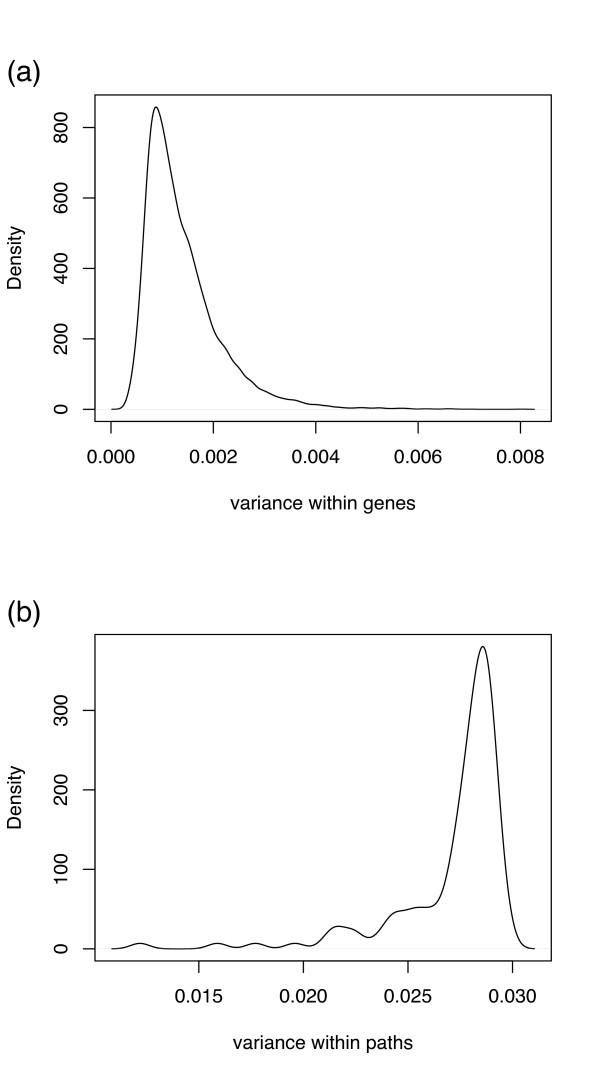
**Variance of *S *by genes/pathways**. Variance in *S *among genes (a) and among pathways (b) in prostate data. Variance within genes is much smaller than in pathways, suggesting that high *S *will tend to be reproduced for other pairs involving the same gene.

It should be noted that the distributions of *S*_GPC _by pathway and by gene are quite dissimilar in the prostate data; consider, for instance, variance of *S*_GPC _within each gene (over all pathways) and within each pathway (over all genes). Distributions of Var(*S|g*) and Var(*S|p*) from the prostate data set are presented in Fig. 5. It is readily apparent that pathways exhibit larger variation in *S*_GPC _than do genes, suggesting that the expression of individual genes, rather than pathways, are typically responsible for the observed loss of correlation. Put another way, if a given gene-pathway pair is implicated in a loss of correlation, it is common for that same gene (but not pathway) to be implicated in other flagged pairs (as is the case in Table 2); in a heat map image of *S *across genes and pathways, this would be exhibited by the appearance of stripes. (A threshold FDR value of 0.05 for differential expression the genes *g *was chosen to ensure that marginal distributions of *g *are similar between phenotypes and limit the number of high *S*_GPC _pairs which are driven by highly significant differential expression of a single gene.)

The biological significance of the flagged genes and pathways are of interest. At the top of the list, *solute carrier family 26 (SLC26A2)*, a membrane sulfate transporter, appears. Evidence exists that *SLC26A2 *may be involved in the sensitivity of tumor cells to chemotherapy [23]. The *FGFR1 oncogene partner (FGFR1OP)*, which occurs 19 times in the top 40 pairs, encodes a largely hydrophilic protein which is thoughy to play a role in proliferation and differentiation [24]. *FGFR1OP *has been found to be a marker for lung cancer progression, but has so far shown little association with prostate cancer [25,26]. *Proprotein convertase subtilisin/kexin type 6 (PCSK6) *is a member of a proprotein convertase family that processes latent precursor proteins into their biologically active products; amongst its targets are TGF*β *proteins, which are considered to have a crucial role in tumorigenesis [24] (while *S*_GPC _is high for the PCSK6-TGF*β *pathway pair (0.79) the loading criteria of 0.6 is not met for the TGF*β *pathway).

A greater diversity of pathways is represented; amongst those which appear multiple times are several biosynthesis pathways, some of which are found to be differentially regulated in other cancers [27], as well as several degradation pathways. For instance, the limonene and pinene degradation pathway, which appears twice, has been suggested by other studies as playing a potential role in prostate cancer [28], and its expression may be sensitive to molecules with anticancer activity [29]. Pathways which are known to be involved in carcinogenesis and cell proliferation, including JAK-STAT signaling, RNA polymerase, and hedgehog pathways, are also represented.

**Figure 6:**
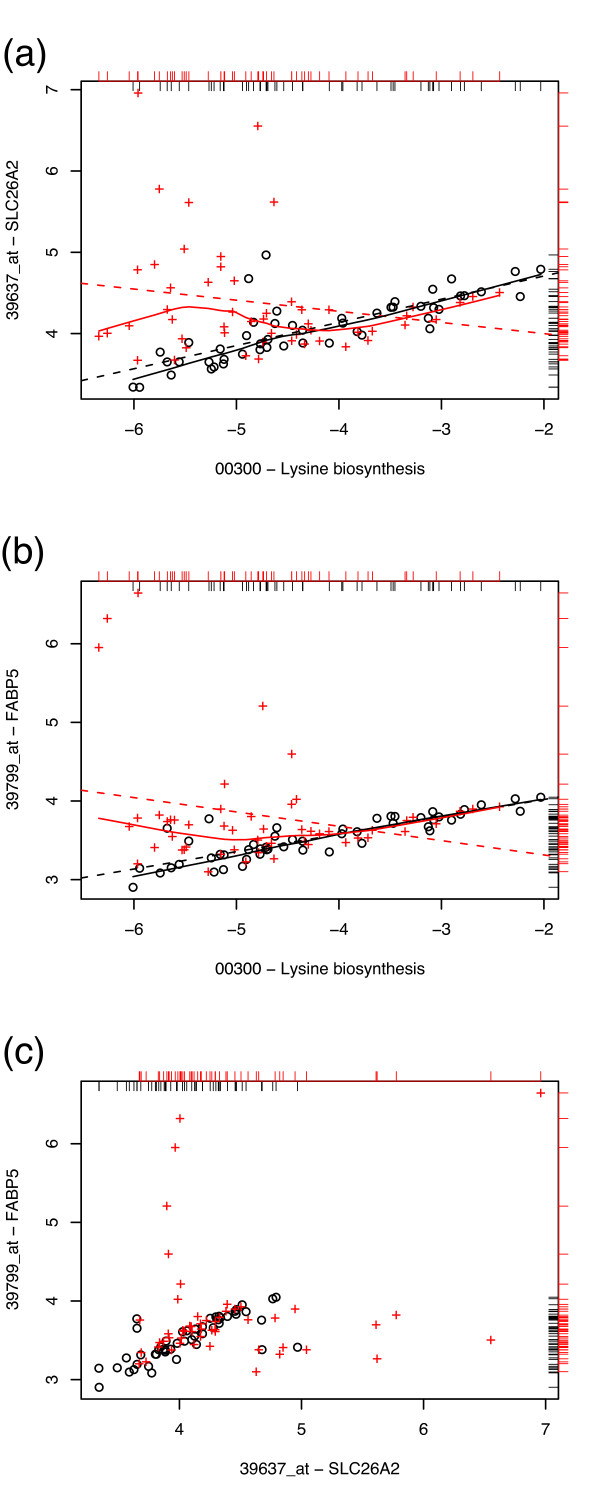
**Decorrelations do not come from the same samples**. Plots of (a) sulfate transporter (SLC26A2) vs. lysine biosythesis pathway; (b) fatty acid binding protein (FABP5) vs. lysine biosythesis pathway; (c) fatty acid binding protein (FABP5) vs. sulfate transporter (SLC26A2). Loess curves and rug plots are given as in Fig. 4. Comparison of the plots reveals that the tumor cell samples responsible for the loss of correlation in sulfate transporter vs. lysine biosynthesis are not those which also exhibit loss of correlation in fatty acid binding protein vs. lysine biosythesis; it is clear from (c) that amongst tumor samples, high levels of expression in one gene do not necessarily correspond to that in the other, suggesting that the outlying points in plots (a, b) do not originate with the same sample.

It may reasonably be asked whether the decorrelated points come from the same samples for all gene-pathway pairs; i.e., if a subset of the samples are outliers by all such criteria. Fig. 6(a,b) provides a suggestion that this is not the case (the points may be compared vertically due to the common *x *axis), and this is readily checked by a joint gene expression plot, Fig. 6(c). As with the gene-path plots, a subset of the tumor samples is observed to behave as the normal samples; in fact, the correlation is similar enough between the tumor and normal samples that CorScore [10] is not particularly high. Yet, it is clear that there exist samples (exclusively from tumor cells) which exhibit particularly high expression levels for one, but not the other, gene; from this we may conclude that the out-of-correlation points do not originate from the same samples for the cases depicted in Fig. 6, and that the mechanisms responsible for overex-pression in one gene are not the same as that in the other.

##### Lung data

**Figure 7:**
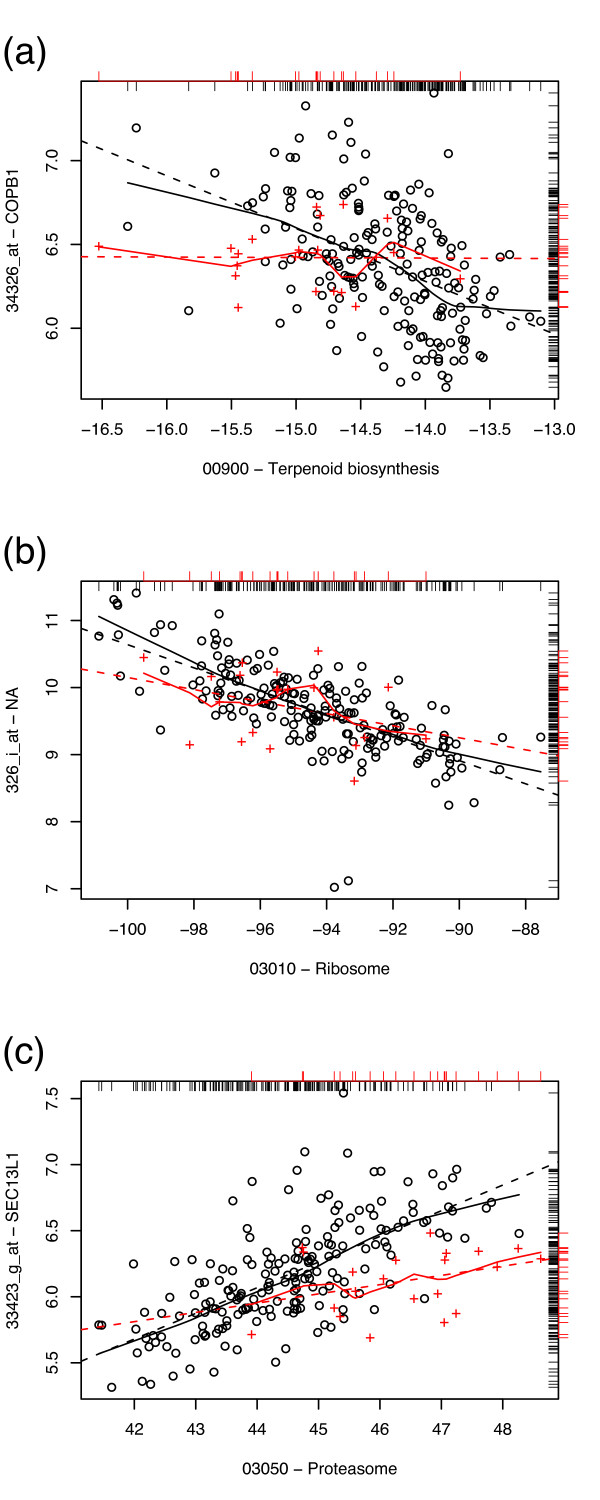
**High *S*_GPC _pairs, lung data**. Sample plots of gene expression vs. pathway expression in adenocarcinoma (black circles) and squamous cell carcinoma (red crosses) of the lung. Loess curves (solid line) and least squares linear fits (dashed line) for the two classes are given as a visual guide. Marginal distributions of the data are given as rug plots (black, inward for adenocarcinoma samples; red, outward for SCC samples). From top to bottom: (a) coatomer protein complex (COPB1) vs. terpenoid biosynthesis pathway (*S*_GPC _= 0.450); (b) Ribosomal protein S20 (RPS20, 326_i_at) vs. ribosome pathway (*S*_GPC _= 0.507); (c) Sec13-like protein (SEC13) vs. proteasome pathway (*S*_GPC _= 0.411).

In the lung cancer data, 113522 gene-pathway pairs were eligible for inclusion, with median *S*_GPC _of 0.17. Twenty-eight pairs at the high end of the *S*_GPC _distribution for the lung data are given in Table 3. Plots exemplifying the loss of correlation seen in the lung data set are given in Fig. 7. The SCC samples appeared to behave as a subset of adenocarcinoma samples; the breadth of the adenocarcinoma samples is unsurprising, given the challenges posed by subclassification within lung adenocarcinomas [15]. As with the prostate data, 10^4 ^resamplings were used for significance testing.

In contrast to the flagged prostate pairs, those from the lung data are dominated by few paths and many genes, most notably the proteasome (which is responsible for protein degradation) and terpenoid biosynthesis. (The Wnt signaling, TGF*β*, and hedgehog pathways did not meet the loading criteria in the lung data.)

**Figure 8:**
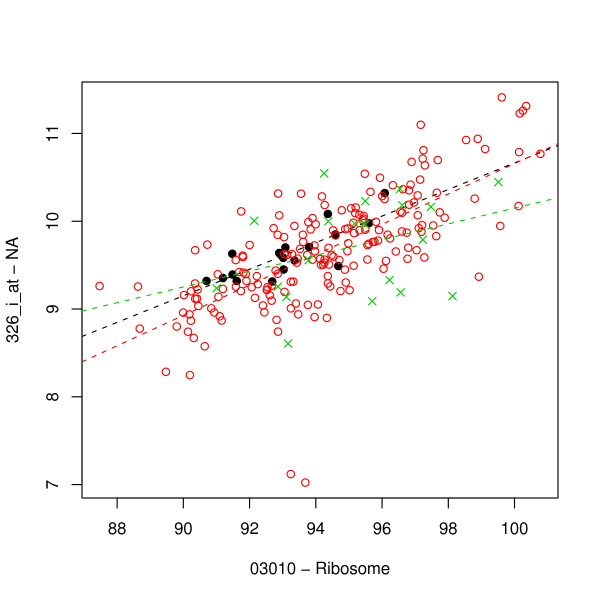
**RPS20 vs. Ribosome pathway summary**. Plot of RPS20 (Affymetrix probe 326_i_at) vs. pathway summary expression for the ribosome pathway (not including RPS20) for normal lung (black filled circles), lung adenocarcinoma (red open circles), and squamous cell carcinoma of the lung (green crosses); dashed lines depict corresponding least-squares linear fits. A statistically significant correlation exists in normal and adenocarcinoma samples, but not in squamous cell carcinoma samples.

Most interestingly, the probe 326_i_at appears in conjunction with the ribosome pathway amongst high-*S*_GPC _pairs. In fact, 326_i_at is a probe for the ribosomal protein *RPS20 *[30] (and hence is part of the ribosome pathway); the absence of this annotation in the Bioconductor HGU95AV2 package permitted its inclusion despite the fact that it fails the *g *∉ *G*_*P *_criterion. As a result, the 326_i_at was treated by the analysis as if it were not part of the ribosome pathway, yet it was clearly identified as a gene of interest with respect to the ribosome pathway, for which the expressions are correlated in normal and adenocarcinoma samples but not in squamous cell carcinoma. As such, the 326_i_at-Ribosome pair represents a strong example of the sort of pairs which this method is designed to identify. The plot given in Fig. 8 shows the statistically significant 326_i_at-Ribosome correlation that is present in adenocarcinoma and normal lung samples, but not in squamous cell carcinoma samples.

## Conclusion

We presented a method for finding relationships between the expression of a gene and that of a known pathway, that are changed across phenotypes. This method defines the expression of a known pathway via a summary value based on principal component analysis. The correlation between the pathway summary expression level and the expression levels of each individual gene is compared across phenotypes to search for interesting gene-pathway pairs. Our approach allows one to efficiently and scalably examine even millions of such pairs. By restricting analysis to pathways for which the first principal components can be meaningfully compared and to genes which do not show differential expression by themselves, higher-order gene interactions may be compared between phenotypes. Gene-pathway pairs for which there is a significant disruption in the correlation across the phenotypes may be flagged. Our approach may be a useful complement to gene-at-a-time analysis; the difference is that in single-gene analyses only the expression of that gene and the phenotype are typically considered, while in ours we consider jointly the gene in question, all the genes in a pathway, and the phenotype.

This procedure was applied to prostate and lung cancer data sets with several promising results. The flagged pairs from this analysis serve as examples of the utility of this method, indicating losses of correlation between gene-pathway pairs for genes which did not exhibit statistically significant differences in single-gene analyses. In addition to suggesting heretofore unknown interactions which could be followed up in biological studies, the method identified a known interaction which had been overlooked in the annotation, suggesting that biologically meaningful associations can be found with this approach.

A concern which follows naturally from these findings is whether, rather than being pathway specific, the pathway summaries may represent trends in the overall dataset. In our implementation, this is an unlikely situation due to the small proportion of genes that are involved in any particular pathway: there are 4252 genes that are associated with any pathway, but the largest pathway meeting the selection criteria in either data set (the Wnt pathway) comprises 184 genes. Accordingly, the projection of the "global" PC1 onto any single pathway is small and any pathway PC1 would be a poor descriptor of trends in the overall dataset. Nonetheless, it is possible that artifacts adding variability to the samples may affect many pathway summaries in similar ways. The risk of this can be reduced by careful normalization and preprocessing.

While the KEGG database [18] was used as a basis for the PCA summarization in our examples, the method can in principle be implemented with any definition of gene groupings for which the first principal component would be a meaningful descriptor. The use of PCA in this method constitutes an improvement over simpler approaches (such as averaging the correlation between the gene of interest and each of those on a given pathway) in that it permits the weighing of pathway genes such that those with greater variation contribute more strongly to the final result.

The use of the first principal component as a summary of pathway expression may be further exploited to examine coordination of pathway activity under different conditions. Measuring pathway-pathway correlations is one such approach, although overlap of pathway components may potentially complicate the interpretation of the results. Another option would be to use a gene set enrichment algorithm [7,31], where, for each pathway *P*, the gene scores *S*_GPC _are used to identify other pathways which are enriched for genes having differential correlation with pathway *P*. This could indicate phenotype dependent pathway coordination.

Future applications of this method could include analysis by cancer grade or prognosis; for gene-pathway pairs which have a clear correlation in normal samples, it may be possible to use deviations from the normal fit as a way of characterizing tumor samples (which could in turn be used as a basis for cluster analysis in much the same way that gene expressions are presently employed). Identifying samples with significant disruption of these relationships may prove valuable in understanding the genetic basis for the observed clinical differences in prostate cancers, possibly opening avenues for more specific therapeutic intervention.

## Abbreviations

PC: Principal Component; PCA: Principal Components Analysis; GPC: Gene/Pathway Correlation; SCC: Squamous Cell Carcinoma.

## Authors' contributions

RB, GP, and LC contributed to the study conception and design. RB carried out the execution, data analysis, and manuscript composition. GP and LC critically reviewed the manuscript.

## Supplementary Material

Additional File 1 **A documented R package, GPCscore, designed to carry out the algorithm described in this manuscript, is available for download and may be installed as a local source package.** See the R documentation [16,17] for package installation instructions. Click here for file
